# 维奈克拉治疗恶性血液病异基因造血干细胞移植后复发25例疗效及安全性

**DOI:** 10.3760/cma.j.issn.0253-2727.2022.07.003

**Published:** 2022-07

**Authors:** 欣 陈, 增艳 刘, 荣莉 张, 卫华 翟, 巧玲 马, 爱明 庞, 栋林 杨, 祎 何, 嘉璘 魏, 四洲 冯, 明哲 韩, 尔烈 姜

**Affiliations:** 中国医学科学院血液病医院（中国医学科学院血液学研究所），实验血液学国家重点实验室，国家血液系统疾病临床医学研究中心，细胞生态海河实验室，天津 300020 State Key Laboratory of Experimental Hematology, National Clinical Research Center for Blood Diseases, Haihe Laboratory of Cell Ecosystem, Institute of Hematology & Blood Diseases Hospital, Chinese Academy of Medical Sciences & Peking Union Medical College, Tianjin 300020, China

**Keywords:** 造血干细胞移植, 维奈克拉, 抢先/挽救治疗, Hematopoietic stem cell transplantation, Venetoclax, Preemptive/salvage therapy

## Abstract

**目的:**

观察维奈克拉（VEN）治疗异基因造血干细胞移植（allo-HSCT）后复发恶性血液病患者的疗效及安全性。

**方法:**

回顾性分析2021年2月至2021年11月在中国医学科学院血液病医院接受VEN治疗的25例allo-HSCT后复发患者的临床资料，其中微小残留病（MRD）阳性患者15例（抢先治疗组），形态学复发患者10例（挽救治疗组）。两组VEN剂量均为400 mg/d，联合唑类抗真菌药物时减量至100 mg/d。

**结果:**

①抢先治疗组男7例，女8例，中位年龄32（18～52）岁；急性髓系白血病（AML）13例，急性淋巴细胞白血病（ALL）1例，原发性骨髓纤维化（PMF）1例；MRD阳性至应用VEN中位时间为2.5（0～12.5）个月。VEN中位疗程数为2（1～4）个。第1疗程治疗第7天VEN中位血药浓度为1 945（688～5 383）µg/L。VEN治疗1疗程后8例患者MRD转为阴性（获得主要反应），4例MRD较治疗前下降50％，3例无效，总体反应率（ORR）为80％（12/15）。9例治疗第7天VEN血药浓度<1 000 µg/L或>3 000 µg/L患者中3例（33.3％）MRD转阴，6例VEN血药浓度1 000～3 000 µg/L患者中5例（83.3％）MRD转阴。5例患者出现3/4级中性粒细胞减少，5例患者出现3/4级血小板减少，无新发严重感染致死病例。②挽救治疗组男7例，女3例，中位年龄44（28～59）岁；AML 6例，ALL 2例，不典型慢性髓性白血病（aCML）1例，骨髓增生异常综合征难治性血细胞减少伴有多系发育异常（MDS-RCMD）1例；复发至应用VEN中位时间为0（0～1）个月；VEN中位疗程数为1（1～2）个；第1疗程治疗第7天VEN中位血药浓度为2 419（1 200～6 155）µg/L。VEN治疗1疗程后3例获得完全缓解（CR）（主要反应），3例获得部分缓解（PR），4例无效，ORR为60％（6/10）。4例治疗第7天VEN血药浓度>3 000 µg/L患者中1例获得CR，6例VEN血药浓度1 000～3 000 µg/L患者中2例获得CR。10例患者均出现3/4级中性粒细胞减少及3/4级血小板减少。1例患者因严重肺感染死亡。③中位随访4.5（1～8.5）个月，抢先治疗组、挽救治疗组总生存（OS）率分别为（70.2±12.7）％、（50.0±15.8）％（*χ*^2^＝1.873，*P*＝0.171）。1疗程VEN治疗获得主要反应、未获得主要反应患者的OS率分别为（90.9±8.7）％、（36.2±14.7）％（*χ*^2^＝6.843，*P*＝0.009）。3例存在TP53突变的患者在VEN治疗后均获得主要反应。

**结论:**

应用VEN对allo-HSCT后复发的恶性血液病患者进行抢先/挽救治疗有一定疗效且耐受性良好，监测VEN血药浓度有望提高疗效。VEN抢先/挽救治疗1个疗程未获得主要反应的患者预后较差，需尽早转换其他治疗方案。

## Abstract (en)

**Objegtive:**

To investigate the efficacy and safety of preemptive/salvage therapy with venetoclax (VEN) in patients with recurrence after allogeneic hematopoietic stem cell transplantation (allo-HSCT).

**Methods:**

Retrospective analysis the clinical data of 25 patients with minimal residual disease (MRD) positive or morphological recurrence after allo-HSCT treated with VEN in the hematological Hospital of Chinese Academy of Medical Sciences from 2021.2 to 2021.11, there were 15 MRD positive patients (preemptive treatment group) and 10 morphological recurrence patients (salvage treatment group). The dose of VEN in both groups was 400 mg/d, which was reduced to 100 mg/d when combined with azole antifungal drugs.

**Results:**

①In the preemptive group, there were 7 males and 8 females, with a median age of 32 (18–52) years; There were 13 cases of acute myeloid leukemia (AML), 1 case of acute lymphoblastic leukemia (ALL) and 1 case of primary myelofibrosis (PMF); the median time from MRD positive to the application of VEN was 2.5 (0–12.5) months. The median course of treatment was 2 (1–4). On the 7th day of the first course of treatment, the median concentration of VEN was 1945 (688–5383) µg/L. After one course of VEN treatment, MRD in 8 patients turned negative (major responses), MRD in 4 patients decreased by 50％ compared with that before treatment, 3 cases were ineffective, and the overall response rate (ORR) was 80％ (12/15). On the 7th day of treatment, 3 of the 9 patients with VEN blood concentration <1 000 µg/L or >3 000 µg/L turned negative for MRD (33.3％), and 5 of the 6 patients with VEN blood concentration between 1000 and 3000 µg/L turned negative for MRD (83.3％). Grade 3/4 neutropenia occurred in 5 patients (33％) and grade 3/4 thrombocytopenia occurred in 5 patients (33％), there were no new cases of severe infection and death. ②In the salvage group, there were 7 males and 3 females, with a median age of 44 (28–59) years; there were 6 cases of AML, 2 cases of ALL, 1 case of atypical chronic myeloid leukemia (aCML), 1 case of refractory hemopenia with multiline dysplasia (MDS-RCMD); the median time from relapse to application of VEN was 0 (0–1) months. The median treatment was 1 (1–2) course. The median concentration of VEN on the 7th day of the first course of treatment was 2 419 (1 200–6 155) µg/L. After one course of VEN treatment, 3 cases achieved complete remission (CR) (major responses) and 3 cases achieved partial remission (PR), 4 cases were ineffective and the ORR was 60％ (6/10). On the 7th day of treatment, 1 of the 4 patients with VEN blood concentration >3 000 µg/L achieved CR (25％), and 2 of the 6 patients with VEN blood concentration between 1 000 and 3 000 µg/L achieved CR (33.3％). Grade 3/4 neutropenia and grade 3/4 thrombocytopenia occurred in 10 patients (100％). One patient died of severe pulmonary infection. ③The median follow-up was 4.5 (1–8.5) months. The overall survival rate (OS) of the preemptive group and the salvage group were (70.2±12.7)％ and (50.0±15.8)％, respectively (*χ*^2^=1.873, *P*=0.171). The OS of patients with and without primary response to one course of VEN were (90.9±8.7)％ and (36.2±14.7)％ respectively (*χ*^2^=6.843, *P*=0.009). Three patients with TP53 mutation achieved the major responses after VEN treatment.

**Conclusion:**

Preemptive/salvage therapy with VEN after allo-HSCT in patients with hematological malignancies is effective and well tolerated, monitoring the concentration of VEN is expected to improve the curative effect. The prognosis of patients who fail to reach the major responses after one course of preemptive/salvage treatment with VEN is poor, so they need to switch to other treatment schemes as soon as possible.

异基因造血干细胞移植（allo-HSCT）是治疗血液系统恶性疾病的有效方法，但移植后复发仍然是死亡的主要原因[1]–[2]。移植后复发患者治疗选择有限，且预后不良[3]。2004年至2008年，在达纳法伯癌症研究所（Dana-Farber Cancer Institute）进行的1 080例移植中，351例复发患者的3年总生存（OS）率仅为19％[4]。对于骨髓增生异常综合征（MDS）和急性髓系白血病（AML）患者，阿扎胞苷和小剂量地西他滨（DAC）联合或不联合供者淋巴细胞输注（DLI）已成为allo-HSCT后复发的常用治疗方法[5]，2年OS率为12％～29％[6]–[10]。强化疗联合DLI与二次移植2年OS率分别为25％、26％，5年OS率分别为15％、19％[11]。

维奈克拉（VEN）是一种选择性小分子BCL-2抑制剂。单药治疗复发AML的总体反应率（ORR）为19％[12]，联合去甲基化药物（HMA）时ORR可达50％～70％[13]–[14]，但治疗恶性血液病患者allo-HSCT后复发鲜有报道。本研究对我中心接受VEN治疗的25例allo-HSCT后复发恶性血液病患者进行回顾性分析，初步观察其疗效与安全性。

## 病例与方法

一、病例

对2021年2月至2021年11月在中国医学科学院血液病医院（中国医学科学院血液学研究所）造血干细胞移植中心接受VEN治疗的25例allo-HSCT后复发患者进行回顾性分析。其中15例患者移植后微小残留病（MRD）阳性，给予VEN抢先治疗（抢先治疗组），10例患者移植后发生形态学复发，给予VEN进行挽救治疗（挽救治疗组）。

抢先治疗组男7例，女8例，中位年龄32（18～52）岁；AML 13例，急性淋巴细胞白血病（ALL）1例，原发性骨髓纤维化（PMF）1例；高危组10例，中危组1例；移植前MRD阳性11例，MRD阴性4例；同胞全相合移植4例，单倍型移植11例。均给予清髓预处理方案。5例患者合并慢性GVHD（cGVHD），分别为轻度2例（皮肤、口腔各1例），中度3例（肝脏、皮肤2例，肝脏、皮肤、肺1例）。移植后2例患者融合基因持续阳性，其余13例患者中位MRD阳性时间为移植后4.5（1～34）个月。复发时均未再进行基因突变检测。

挽救治疗组男7例，女3例，中位年龄44（28～59）岁；AML 6例，ALL 2例，不典型慢性髓性白血病（aCML）1例，难治性血细胞减少伴有多系发育异常（MDS-RCMD）1例；高危组7例；移植前MRD阳性4例，未缓解（NR）1例；同胞全相合移植8例，单倍型移植2例；均给予清髓预处理方案；均无明显GVHD表现。中位形态学复发时间为移植后10.0（2.5～132.0）个月。复发时1例患者在原有RUNX1、U2AF1突变基础上新增ASXL1、NRAS突变，另有1例新增PRPF8、ASXL1、RUNX1突变，其余患者复发时未再进行基因突变检测。

25例患者中，共有5例复发时合并急性GVHD（aGVHD）/cGVHD，移植后2个月内复发2例，均不适合DLI治疗。5例患者复发时无冻存干细胞，无法行DLI治疗。6例患者应用VEN前已应用过1～3次DLI治疗无效，因此选用VEN治疗。6例患者在VEN治疗1个疗程评估疗效后联合DLI治疗，1例应用VEN联合DLI治疗。

6例伴FLT3突变患者，3例移植前曾应用索拉非尼治疗疗效欠佳（FLT3/TKD、FLT3/JM点突变、T-ALL合并FLT3/ITD各1例），因此复发时均未选用索拉非尼，且当时吉瑞替尼尚未上市，因此也未应用。

二、随访

患者生存情况来自电话随访、查阅门诊/住院病历。随访截止时间为2021年11月5日，中位随访时间为4.5（1.0～8.5）个月。OS定义为从VEN开始治疗至任何原因死亡或随访终止时间。

三、统计学处理

统计分析使用SPSS 16.0软件，采用Kaplan-Meier法进行生存分析，组间比较采用Log-rank检验。*P*<0.05为差异有统计学意义。

## 结果

一、第1疗程VEN疗效

抢先治疗组MRD阳性至应用VEN中位时间为2.5（0～12.5）个月。VEN剂量400 mg/d（联合唑类抗真菌药物时减量至100 mg/d），第1疗程治疗第7天VEN中位血药浓度为1 945（688～5 383）µg/L，血药浓度<1 000 µg/L增加VEN剂量，>3 000 µg/L时减少VEN剂量。VEN治疗1个疗程后，15例患者中8例MRD转为阴性（获得主要反应），4例MRD较治疗前下降50％，3例无效（NR），ORR为80.0％（12/15）。MRD阳性至应用VEN时间≤3个月的9例患者中6例（66.7％）MRD转阴，>3个月的6例患者中2例（33.3％）MRD转阴；9例治疗第7天VEN血药浓度<1 000 µg/L或>3 000 µg/L患者中3例（33.3％）MRD转阴，6例VEN血药浓度1 000～3 000 µg/L患者中5例（83.3％）MRD转阴。

挽救治疗组复发至应用VEN中位时间为0（0～1）个月。VEN剂量400 mg/d（联合唑类抗真菌药物减量至100 mg/d），第1疗程治疗第7天VEN中位血药浓度为2 419（1 200～6 155）µg/L，>3 000 µg/L时减少VEN剂量。VEN治疗1疗程后，3例完全缓解（CR）（获得主要反应），3例部分缓解（PR），4例NR，ORR为60％（6/10）。8例复发后立即给予VEN治疗的患者中3例（37.5％）获得CR，2例间隔1个月后应用的患者均未获得CR。4例治疗第7天VEN血药浓度>3 000 µg/L患者中1例（25.0％）获得CR，6例VEN血药浓度1 000～3 000 µg/L患者中2例（33.3％）获得CR。

3例存在TP53突变的患者VEN治疗后均获得主要反应；6例存在FLT3突变的患者中仅1例获得主要反应；4 例MLL-AF10阳性、2例TLS-ERG阳性患者治疗后均未获得主要反应（表1）。

**表1:** 25例接受VEN治疗异基因造血干细胞移植后复发恶性血液病患者的临床特征及疗效

例号	性别	年龄（岁）	原发病诊断	移植类型	移植前高危突变/融合基因	复发至应用VEN时间（月）	治疗第7天VEN血药浓度（µg/L）	VEN第1疗程疗效	VEN疗程	随访时间（月）	随访结束时骨髓状态	转归（死亡原因）
1	女	46	AML-M5	Haplo	FLT3/ITD	2.5	945	MRD转阴（主要反应）	4	6	MRD阴性	存活
2	男	31	AML-M2b	MSD	未检出	3	1510	MRD转阴（主要反应）	2	5	MRD阴性	存活
3	男	52	AML(aCML转化）	Haplo	ASXL1	0	3580	MRD转阴（主要反应）	3	6	MRD阴性	存活
4	男	32	AML-M4EO	Haplo	未检出	3	1784	MRD转阴（主要反应）	4	6	MRD阴性	存活
5	男	27	T-ALL	Haplo	TP53	0	5238	MRD转阴（主要反应）	1	3	MRD阴性、髓外复发	死亡（复发）
6	女	51	AML（MDS转化）	MSD	TP53	2	1945	MRD转阴（主要反应）	4	8	MRD阴性	存活
7	女	31	AML-M2b	Haplo	KIT	12.5	2298	MRD转阴（主要反应）	3	3.5	MRD阴性	存活
8	女	49	AML-M4	MSD	NUP98-HOXA9	8.5	1440	MRD转阴（主要反应）	1	7.5	形态学复发	存活
9	女	38	AML-M5	Haplo	MLL-AF10	0	1417	MRD下降	2	4	形态学复发	存活
10	男	18	PMF	Haplo	未检出	5	3789	MRD下降	1	3	形态学复发	死亡（复发）
11	女	46	AML-M5	Haplo	TLS-ERG	0	842	MRD下降	3	6	MRD阳性（升高）	存活
12	女	43	AML-M4EO	Haplo	FLT3/JM点突变	5	5383	MRD下降	3	5	MRD阴性	存活
13	男	27	AML-M5	Haplo	TLS-ERG	10	688	无效	1	1	形态学复发	死亡（复发）
14	男	32	AML-M2b	Haplo	FLT3/ITD	0	3222	无效	2	5	形态学复发	死亡（复发）
15	女	29	AML-M2b	MSD	未检出	5.5	3171	无效	1	2.5	形态学复发、髓外复发	存活
16	男	59	MDS-RCMD	MSD	未检出	0	6155	CR（主要反应），MRD阴性	2	7	MRD阴性	存活
17	女	28	B-ALL	MSD	未检出	0	1551	CR（主要反应），MRD阳性	1	4.5	MRD阴性	存活
18	女	33	AML-M5	MSD	TP53	0	1442	CR（主要反应），MRD阳性	1	8.5	MRD阴性	存活
19	男	44	T-ALL	MSD	FLT3/ITD	1	4068	PR	1	1	MRD阴性	死亡（肺感染）
20	男	57	AML-M5	MSD	MLL-AF10	0	2886	PR	1	2	形态学复发	死亡（复发）
21	女	35	AML-M5	Haplo	MLL-AF10	0	3198	PR	1	5	形态学复发	存活
22	男	49	AML-M5	MSD	FLT3/ITD	0	1952	NR	1	1.5	NR	死亡（复发）
23	男	47	aCML	MSD	FLT3/TKD	0	1200	NR	1	4	NR	存活
24	男	44	AML-M5	Haplo	未检出	0	1747	NR	1	1.5	NR	死亡（复发）
25	男	37	AML-M2	MSD	MLL-AF10	1	3423	NR	1	3	NR	死亡（复发）

注：VEN：维奈克拉；AML：急性髓系白血病；aCML：不典型慢性髓性白血病；ALL：急性淋巴细胞白血病；MDS：骨髓增生异常综合征；PMF：原发性骨髓纤维化；MDS-RCMD：难治性血细胞减少伴有多系发育异常；Haplo：单倍型供者；MSD：同胞全相合供者；MRD：微小残留病；CR：完全缓解：PR：部分缓解；NR：未缓解。例1～15为抢先治疗（allo-HSCT后MRD阳性），例16～25为挽救治疗（allo-HSCT后形态学复发）

二、VEN总体疗效

抢先治疗组VEN治疗中位疗程为2（1～4）个。中位随访5（1～8）个月，8例1疗程MRD转阴患者中5例MRD持续阴性，1例第2周期VEN后MRD转为阳性，改为阿扎胞苷联合干扰素治疗后MRD再次转阴，上述6例患者目前均MRD阴性存活。1例患者1疗程后自行停药，5个月后形态学复发，目前带病存活。另1例骨髓MRD持续阴性，但发生髓外（中枢神经系统、颈部淋巴结）复发而死亡。4例1疗程MRD下降患者中，1例经3周期VEN后MRD转阴；1例治疗3周期后MRD上升，改为化疗联合DLI治疗，目前仍存活；2例在治疗2个月后形态学复发，1例死亡，1例CAR-T细胞治疗中。3例无效患者均形态学复发，2例死亡，1例拟行二次移植。

挽救治疗组VEN治疗中位疗程1（1～2）个。中位随访3.5（1.0～8.5）个月，3例CR患者后期均联合其他（化疗+DLI或CAR-T）治疗，目前均持续CR存活。3例PR患者，1例VEN后联合DLI，抑制期因肺部感染死亡；2例VEN后联合化疗或DLI，再次出现形态学复发，1例放弃治疗死亡，1例正在治疗中。4例NR患者，虽然随后进行化疗、DLI、CAR-T、程序性死亡受体1（PD-1）单抗等治疗，均未达PR，最终3例死亡，1例目前仍在PD-1单抗治疗中。

三、不良反应

抢先治疗组患者未发生肿瘤溶解综合征，5例（33％）患者出现3/4级中性粒细胞减少，5例（33％）患者出现3/4级血小板减少。3例患者出现无明显感染灶的发热，抗感染治疗后体温控制；2例患者出现口腔溃疡，1例患者出现肺感染，1例患者出现带状疱疹，治疗后均好转。6例治疗第7天VEN浓度>3 000 µg/L的患者中，发生3/4级中性粒细胞减少4例次、3/4级血小板减少4例次；无新发严重感染致死病例；无aGVHD发生；肝肾功能无明显异常。

挽救治疗组患者亦未发生肿瘤溶解综合征，10例（100％）患者均出现3/4级中性粒细胞减少及3/4级血小板减少。4例患者出现无明显感染灶的粒细胞缺乏伴发热，抗感染治疗后体温控制；肺感染、大肠埃希菌菌血症患者各1例，治疗后好转；1例患者在应用VEN前即发生严重肺感染，VEN治疗后肺感染加重并死亡。治疗第7天VEN浓度>3 000 µg/L的4例患者中3例合并感染（1例死于肺感染）；VEN挽救治疗后无aGVHD发生；肝肾功能无明显异常。

四、转归

至随访截止，抢先治疗组15例患者中死亡4例，挽救治疗组10例患者中死亡5例。抢先治疗组、挽救治疗组OS率分别为（70.2±12.7）％、（50.0±15.8）％（*χ*^2^＝1.873，*P*＝0.171）。1疗程VEN治疗达主要反应（抢先治疗组MRD转阴，挽救治疗组CR）、未达主要反应患者OS率分别为（90.9±8.7）％、（36.2±14.7）％（*χ*^2^＝6.843，*P*＝0.009）。

## 讨论

allo-HSCT是恶性血液系统疾病患者的一种有效的治疗选择，其治疗失败的最常见原因为复发，复发后治疗仍然是一个巨大的挑战[15]。迄今为止，对于allo-HSCT后复发的患者没有标准的治疗方案[16]。常用治疗策略为DLI、HMA、强化疗或二次移植。虽然这些方法在某些患者中取得了疗效，但无法实现持续CR和长期生存[17]。VEN是一种口服BCL-2抑制剂，通过直接与BCL-2蛋白结合，取代促凋亡蛋白，从而恢复凋亡过程，在过度表达BCL-2的肿瘤细胞中显示出细胞毒活性[13],[18]–[19]并触发线粒体外膜通透化，释放细胞色素C来诱导凋亡[14]。VEN在高危MDS及复发难治AML中的疗效令人鼓舞，也为allo-HSCT后复发患者的治疗带来新的治疗手段。2020年ASH一项研究收集了PubMed、Cochrane、Embase和Science网中的4项临床试验（134例）和18项观察研究（756例），对移植后复发AML/MDS患者进行的各项挽救治疗进行有效性和安全性评估，结果显示：二次移植后74％～84％的患者获得CR，但最终15％的患者获得持续CR，44％的患者再次复发，68个月的OS率为14％。阿扎胞苷联合索拉非尼的试验（纳入8例患者）中，有效率为50％，1年OS率为31％。阿扎胞苷单药治疗的有效率为13％～32％（256例）。在3项观察性研究（纳入48例患者）中，应用VEN后42％～53％的患者获得CR，43％的患者维持疗效≥3个月。Schuler等[17]回顾性收集了来自11个德国移植中心的32例髓系肿瘤患者HSCT复发后挽救治疗的资料，VEN与阿扎胞苷（13例）或地西他滨（DAC）（19例）联用，ORR为47％，平均随访8.4个月，25例（78％）死亡，7例存活。预期中位OS期为3.7个月。此外，VEN还被发现在临床上对多种血液系统恶性肿瘤有效，包括多发性骨髓瘤、MDS、ALL等[20]。Pullarkat等[21]报道了VEN与低剂量navitoclax（一种BCL-XL/BCL2抑制剂）联合使用，治疗复发/难治（R/R）ALL或淋巴母细胞淋巴瘤的儿童和成人（包括之前接受过allo-HSCT或免疫治疗）患者，耐受性良好，CR率为60％。Richter等[22]研究证明了Pan-AKT抑制剂MK-2206能有效阻止B-ALL细胞增殖，并首次描述了MK-2206和VEN联合治疗B-ALL的协同效应。Richard-Carpentier等[23]应用VEN联合化疗治疗13例中位年龄46（20～75）岁的R/R T-ALL患者，在10例可评估疗效的患者中6例获得CR，其中3例血液学完全缓解。中位OS期、无复发生存期分别为7.7个月、4.0个月。Gangat等[24]回顾性分析了多项研究，结果显示VEN在高危MDS（治疗初期CR率39％，HMA失败后CR率5％～14％）和骨髓增殖性肿瘤（MPN）急变期（CR率25％）中也显示出疗效。在本研究，25例患者疾病类型包括AML、ALL、MDS、PMF及aCML，高危组患者17例，移植前MRD阳性患者15例，移植后MRD阳性/形态学复发应用VEN治疗1疗程可达较好疗效：抢先治疗组ORR为80％，挽救治疗组为60％。中位随访4.5（1.0～8.5）个月，抢先治疗组患者死亡4例，OS率为（70.2±12.7）％，截止随访日期，尚未达到中位生存时间；挽救治疗组患者死亡5例，OS率为（50.0±15.8）％，中位生存时间为3个月。1疗程VEN治疗达主要反应患者OS率为（90.9±8.7）％，未达主要反应患者OS率为（36.2±14.7）％，差异有统计学意义（*P*＝0.009）。抢先治疗组MRD阳性至应用VEN时间≤3个月的9例患者中6例（66.7％）MRD转阴，>3个月的6例患者中2例（33.3％）MRD转阴。我们的研究结果显示：恶性血液病患者allo-HSCT后复发应用VEN进行抢先治疗疗效优于挽救治疗，且抢先治疗时早期加用VEN有助于提高疗效，1疗程VEN治疗达主要反应患者OS显著优于未达主要反应患者（*P*＝0.009）。因此allo-HSCT后需重视MRD监测，MRD阳性后早期加用VEN进行抢先治疗，提高疗效。应用VEN进行1疗程抢先/挽救治疗未达主要反应患者预后差，需尽早转换其他方案治疗，达主要反应患者预后良好，可继续VEN或联合DLI等其他治疗。

我们在本研究中发现，VEN常规应用剂量均为400 mg/d（联合唑类抗真菌药物减量至100 mg/d），抢先治疗组治疗第7天VEN血药浓度<1 000 µg/L或>3 000 µg/L 9例患者中3例MRD转为阴性，VEN血药浓度1 000～3 000 µg/L 6例患者中5例MRD转为阴性。治疗第7天VEN血药浓度>3 000 µg/L 6例患者，4例出现3/4级中性粒细胞减少，4例出现3/4级血小板减少；挽救治疗组治疗第7天VEN血药浓度>3 000 µg/L 4例患者中1例获得CR，VEN血药浓度1 000～3 000 µg/L 6例患者中2例获得CR。治疗第7天VEN浓度>3 000 µg/L 4例患者中3例合并感染，并因肺感染死亡1例。虽然我们根据VEN治疗第7天血药浓度调整剂量，患者VEN治疗后未出现严重感染致死病例（1例治疗前存在严重肺感染患者治疗后死亡除外），但血药浓度>3 000 µg/L患者因骨髓抑制明显导致疗程缩短而影响疗效，浓度<1 000 µg/L患者疗效欠佳，因此维持药物浓度1 000～3 000 µg/L有望提高VEN疗效。

在我们的研究中3例存在TP53突变的患者，应用VEN治疗均达主要反应，与文献[25]报道的1例在移植后复发时出现TP53突变患者，通过VEN治疗达到良好疗效类似，这可能与VEN在高危MDS患者中显示出潜在的诱导凋亡作用，而不依赖于ASXL1、RUNX1、TP53或EZH2等不良突变的存在[26]–[27]有关。根据数据显示，FLT3突变的AML患者对VEN的反应率较低[14],[28]，但也有文献报道VEN治疗FLT3突变患者疗效良好：Guerra等[29]介绍了一项Ⅰb/Ⅱ期临床研究评估VEN与小剂量阿糖胞苷（LDAC）联合治疗不适合强化治疗的老年AML患者，FLT3突变患者的CR/伴骨髓恢复不完全的完全缓解（CRi）为44％。DiNardo等[13]报道了一项多中心Ⅰb期研究，VEN与HMA联合应用于无法接受强化治疗的新诊断AML老年患者，FLT3突变患者的CR/CRi为72％，FLT3突变患者的缓解持续时间为11个月。我们的研究结果显示，6例存在FLT3突变患者，仅1例达主要反应，显示出VEN在移植后抢先/挽救治疗中针对FLT3的低反应率。此外4 例MLL-AF10阳性、2例TLS-ERG阳性患者治疗后均未达主要反应，也预示VEN对这些融合基因阳性患者的治疗效果可能偏差，但需要大宗系列研究以证实。

VEN作用恶性血液病患者移植后复发抢先/挽救治疗的安全性方面，本研究结果和文献[17],[25]报道类似：治疗过程中均未发生肿瘤溶解综合征，无明显肝肾功能损害，无明显GVHD出现。VEN治疗后最常见副反应为骨髓抑制，抢先治疗组5例（33％）患者出现3/4级中性粒细胞减少，5例（33％）患者出现3/4级血小板减少。挽救治疗组10例（100％）患者均出现3/4级中性粒细胞减少及3/4级血小板减少，但VEN治疗后感染均可控，挽救治疗组1例因肺感染死亡，但此患者在应用VEN前即发生严重肺感染，VEN治疗后肺感染加重最终死亡。

本组病例结果显示，应用VEN对 allo-HSCT后复发的恶性血液病患者进行抢先/挽救治疗有一定疗效且耐受性良好，监测VEN浓度有望提高疗效；应用VEN进行1个疗程抢先/挽救治疗未达主要反应患者预后差，需尽早转换其他治疗方案；VEN抢先/挽救治疗可克服TP53突变，但对FLT3突变、融合基因MLL-AF10及TLS-ERG反应较差。上述结果仍需大宗系列研究加以验证。
